# Supplementary material for: Analysis of PPARγ Signaling Activity in Psoriasis
Source: Int J Mol Sci. 2021 Aug 10;22(16):8603. doi: 10.3390/ijms22168603 (PMC8395241; doi:10.3390/ijms22168603)
Supplement: Supplementary file 1 [file ijms-22-08603-s001.zip › Supplemental materials_Analysis of PPARg signaling activity in psoriasis/Pathway models/Models images and html files/Interleukin-17 and interleukin-22 signaling in psoriasis/6347.html]

CXCL8


# Protein CXCL8

|  |  |
| --- | --- |
| URN | urn:agi-llid:3576 |
| Total Entities | 0 |
| Connectivity | 13044 |
| Name | CXCL8 |
| Description | chemokine (C-X-C motif) ligand 8 |
| Notes | The protein encoded by this gene is a member of the CXC chemokine family and is a major mediator of the inflammatory response. The encoded protein is secreted primarily by neutrophils, where it serves as a chemotactic factor by guiding the neutrophils to the site of infection. This chemokine is also a potent angiogenic factor. This gene is believed to play a role in the pathogenesis of bronchiolitis, a common respiratory tract disease caused by viral infection. This gene and other members of the CXC chemokine gene family form a gene cluster in a region of chromosome 4q. [provided by RefSeq, Aug 2017] |
| Primary Cell Localization | Extracellular |
| Class | Ligand |

---

|  |  |
| --- | --- |
| Pathway | Proteins Involved in Stem Cell Exhaustion in Aging |
|  | ECL-cell: CHGA and Histamine Synthesis |
|  | Cortisol in Resolving Inflammation |
|  | Toll-like Receptors Act through MYD88-TIRAP Signaling |
|  | TLR4 Signaling in Leukocytes |
|  | T-Cell Maturation (Hypothesis) |
|  | CXC Chemokine Receptor Signaling |
|  | Platelet Activation via Adhesion Molecules |
|  | Proplatelet Maturation |
|  | Toll-like Receptors in Sterile Inflammation |
|  | Lipoxin A4/FPR2-Related Neutrophil Depression |
|  | MC1R in anti-Inflammatory Signaling |
|  | Find me Signal: Apoptotic Cell Attracts Phagocyte |
|  | Lipids Enhance Apoptotic Cell Engulfment and Reduce Inflammation |
|  | Keep out Signal: LTF Inhibits Neutrophil Recruitment and Inflammation |
|  | Leukotriene Effect on Vascular Endothelial Cell Response |
|  | Eosinophil Survival by Cytokine Signaling |
|  | Eosinophil Activation |
|  | Mast-Cell Activation without Degranulation through TNFSF8 Signaling |
|  | Mast-Cell Activation via IgE Signaling |
|  | Mast-Cell Activation without Degranulation through CYSLTR1/CYSLTR2 Signaling |
|  | Mast-Cell Activation without Degranulation through CXCR4 Signaling |
|  | Mast-Cell Activation without Degranulation |
|  | Mast-Cells De Novo Synthesized Mediators via IgE Independent Signaling |
|  | Mast-Cell Activation without Degranulation through IL1R1 and TLR Signaling |
|  | Neutrophil Recruitment and Priming |
|  | Neutrophil Degranulation via FPR1 Signaling |
|  | Neutrophil Chemotaxis |
|  | Neutrophil Activation via Adherence on Endothelial Cells |
|  | Vascular Endothelial Cell Activation by Blood Coagulation Factors |
|  | Nociception Expression Targets Signaling |
|  | NTSR1 Expression Targets |
|  | BDKRB1/2 -> Interleukins Production |
|  | PTGER2/3 -> Inflammation-Related Expression Targets |
|  | TACR1 -> TNF/IL6/IL8 Production |
|  | IL6 Expression Targets -> Nociception |
|  | IL1B Expression Targets -> Nociception |
|  | CHRNA7 -> IL8 Production |
|  | P2RY11/13/14 -> IL8/10 Production |
|  | Proteins Involved in Atherosclerosis |
|  | Scavenger Receptor OLR1 in Inflammation Related Endothelial Dysfunction |
|  | Dendritic Cells Function in Atherosclerosis |
|  | Proteins Involved in Arterial Hypertension |
|  | Arterial Hypertension |
|  | Proteins Involved in Myocardial Ischemia |
|  | Toll-like Receptors (TLR) in Antiviral Innate Immune Response |
|  | Proteins Involved in Acne Vulgaris |
|  | Inflammatory Reaction in Acne Vulgaris |
|  | Mast-Cells Activation in Atopic Dermatitis |
|  | Proteins with Altered Expression in Melanoma |
|  | Melanoma |
|  | Proteins Involved in Melanoma |
|  | IL17 Signaling in Psoriasis |
|  | Proteins Involved in Psoriasis |
|  | Oxidative Stress in Vitiligo |
|  | Proteins with Altered Expression in Vitiligo |
|  | Vitiligo |
|  | T-Cell Cytotoxic Response against Melanocytes in Vitiligo |
|  | Proteins Involved in Diabetic Nephropathy |
|  | Hepatic Stellate Cells in non-Alcoholic Fatty Liver Disease |
|  | Proteins Involved in non-Alcoholic Fatty Liver Disease |
|  | Proteins Involved in Obesity |
|  | Proteins Involved in Colorectal Neoplasms |
|  | Metastatic Colorectal Cancer |
|  | Paneth-Cell Function in Crohn's Disease |
|  | Proteins Involved in Inflammatory Bowel Diseases |
|  | OipA Signaling in Helicobacter Pylori Infection |
|  | Proteins Involved in Helicobacter Infections |
|  | CagA Phosphorylation Independent Signaling |
|  | Leukocyte Migration toward the Endothelial Cell in Ulcerative Colitis |
|  | Proteins Involved in Ulcerative Colitis |
|  | Antiphospholipid Antibodies in Endothelial Cell |
|  | Hodgkin and Reed-Sternberg Cells in Hodgkin Lymphoma |
|  | Hodgkin Lymphoma |
|  | TNFR/NF-kB Classical Pathway in Multiple Myeloma Plasma Cells |
|  | IL6/IGF1/VEGFA Signaling in Multiple Myeloma |
|  | NOTCH Signaling in Multiple Myeloma |
|  | TNFR/NF-kB Alternative Pathway in Multiple Myeloma Plasma Cells |
|  | Antiphospholipid Antibodies and Endothelial Cell Interaction |
|  | SERPINA1 Associated Liver Damage |
|  | Proteins Involved in Pancreatic Cancer |
|  | TRPM7/8 in Pancreatic Adenocarcinoma |
|  | Eosinophil Survival in Asthma |
|  | Il17 Signaling Related Neutrophilia in Asthma |
|  | Neutrophil Degranulation via FPR1/IL8 |
|  | Mast-Cell Activation in Asthma |
|  | CFTR Facilitates Oxidative Stress in Airway Epithelium in CF |
|  | Proteins Involved in Cystic Fibrosis |
|  | Airway Epithelial Cell Dysfunction |
|  | CFTR Related Ion-Channel Dysfunction in Cystic Fibrosis Airway Epithelium (Class III Mutations) |
|  | Proteins Involved in Gout |
|  | Proteins with Altered Expression in Gout |
|  | Neutrophil Recruitment in Sinovium in Gout |
|  | Monocytes Function in Gout |
|  | Proteins Involved in Osteoarthritis |
|  | Synovial Fibroblast Activation in Psoriatic Arthritis |
|  | Keratinocyte Activation in Psoriatic Arthritis |
|  | Synovial Fibroblast Activation by Citokines in Rheumatoid Arthritis |
|  | TLR2 Induced Synovial Fibroblast Activation in Rheumatoid Arthritis |
|  | Proteins Involved in Rheumatoid Arthritis |
|  | FAS Mediated Apoptosis in Systemic Scleroderma |
|  | Genes with Mutations Associated with Systemic Scleroderma |
|  | Proteins Involved in Systemic Scleroderma |
|  | Toll-like Receptors (TLR) in Periodontitis |
|  | Diabetes Induced Periodontitis |
|  | Estrogen Effects on Pregnancy Gingivitis |
|  | Proteins Involved in Periodontitis |
|  | IL36 Receptor Antagonist (IL1F5) Deficiency (DITRA) |
|  | NF-kB Signaling Activation in Huntington Disease |
|  | Proteins with Altered Expression in Psoriatic Arthritis |
|  | Proteins with Altered Expression in Rheumatoid Arthritis |
|  | Proteins Involved in Ependymoma |
|  | Proteins Involved in Glioma |
|  | Proteins Involved in Adhesion, Migration and Invasion of Endometriotic Cell |
|  | Proteins Involved in Endometriosis |
|  | Coagulation Factors and PAR1/2 Receptors (F2R/F2RL1) in Endometriosis |
|  | Proteins Involved in Cholesteatoma |
|  | HMGB1/RAGE Signaling in Middle Ear Cholesteatoma |
|  | Cholesteatoma |
|  | HPV E6 and E7 Induced Disruption of TNFR/NF-kB Signaling in Keratinocytes |
|  | Proteins Involved in Age-Related Macular Degeneration |
|  | ER Stress in Macular Degeneration |
|  | Proteins Involved in Otitis Media |
|  | Cytokines Trigger Otitis Media |
|  | Proteins with Altered Expression in Asthma |
|  | Alveolar Macrophage Negative Regulation Declined in COPD |
|  | Alveolar Macrophages Dysfunction in COPD |
|  | Glucocorticoid Resistance |
|  | Proteins Involved in Chronic Obstructive Pulmonary Disease |
|  | Alveolar Epithelial Cell Dysfunction in COPD |
|  | Proteins Involved in Chronic Bronchitis |
|  | Genes with Mutations Associated with Cystic Fibrosis |
|  | Proteins Involved in Glomerulonephritis |
|  | Endothelial Cell Dysfunction in Glomerulonephritis |
|  | Proteins Involved in Pyelonephritis |
|  | Proteins with Altered Expression in Pyelonephritis |
|  | Toll-like Receptors in Pyelonephritis |
|  | Endothelial Cell Dysfunction in Pyelonephritis |
|  | AREG -> CREB/CREBBP Expression Targets |
|  | AREG -> NCOR2 Expression Targets |
|  | AREG -> CTNN Expression Targets |
|  | AREG -> HIF1A Expression Targets |
|  | AREG -> AP-1 Expression Targets |
|  | Noradrenaline/Gs Expression Targets |
|  | Dopamine/Gs Expression Targets |
|  | Noreadrenaline/Gq Expression Targets |
|  | Epinephrine/Gs Expression Targets |
|  | Epinephrine/Gq Expression Targets |
|  | Dopamine/Gi Expression Targets |
|  | Elevated Receptors -> Expression Targets in Joint |
|  | Elevated Receptors -> Expression Targets in Bone and Joint |
|  | Elevated Receptors -> Expression Targets in Blood vessel |
|  | Elevated Receptors -> Expression Targets in Blood Vessel |
|  | CXCL6 Expression Targets |
|  | CXCL2 Expression Targets |
|  | CXCL5 Expression Targets |
|  | CXCL12 Expression Targets |
|  | CCL3 Expression Targets |
|  | IL8 Expression Targets |
|  | CSF3 Expression Targets |
|  | CSF2 -> NF-kB Expression Targets |
|  | CSF2 -> STAT Expression Targets |
|  | LIF Expression Targets |
|  | EGF -> CTNN Expression Targets |
|  | EGF -> AP-1/ATF Expression Targets |
|  | EGF -> CREB/CREBBP/ELK/SRF/MYC Expression Targets |
|  | EGF -> MEF/MYOD/NFATC Expression Targets |
|  | EGF -> STAT Expression Targets |
|  | PECAM1 Expression Targets |
|  | F2 -> AP-1/CREB/ELK/SRF/SP1 Expression Targets |
|  | F2 -> STAT1/NF-kB Expression Targets |
|  | PLG -> AP-1/CREB/ELK/SRF/SP1 Expression Targets |
|  | PLG -> STAT1/NF-kB Expression Targets |
|  | FGF18 -> STAT Expression Targets |
|  | FGF18 -> AP-1/CREB Expression Targets |
|  | FGF1 -> STAT Expression Targets |
|  | Elevated Receptors -> Expression Targets in Mouth |
|  | Elevated Receptors -> Expression Targets in Colon |
|  | HBEGF -> STAT Expression Targets |
|  | HBEGF -> AP-1/ATF Expression Targets |
|  | HGF -> STAT Expression Targets |
|  | HGF -> AP-1/CREB/ELK/SRF/MYC Expression Targets |
|  | EGFR Transactivation in Cancer and Non-Cancer Cells |
|  | TAM Receptor Signaling Activation in Cancer |
|  | ANGPT/TEK Maintains Endothelial Cell Quiescence in Cancer |
|  | Lactate as a Signaling Molecule in Cancer Cells |
|  | Proteins with Altered Expression in Cancer Metabolic Reprogramming |
|  | Tumor Infiltrating Macrophages in Cancer Progression and Immune Escape |
|  | IGF1 -> MEF/MYOD/MYOG Expression Targets |
|  | IGF1 -> STAT Expression Targets |
|  | IGF1 -> ELK/SRF/HIF1A/MYC/SREBF Expression Targets |
|  | Fibronectin Expression Targets |
|  | CTGF -> AP-1/CREB/MYC Expression Targets |
|  | ICAM1 Expression Targets |
|  | Fibrinogen Expression Targets |
|  | EGFR -> Expression Targets in Skin |
|  | IFNB1/IFNR Expression Targets |
|  | IFNG/IFNR Expression Targets |
|  | IL7 Expression Targets |
|  | IL13 Expression Targets |
|  | IL15 Expression Targets |
|  | IL3 Expression Targets |
|  | IL10 Expression Targets |
|  | IL11 Expression Targets |
|  | IL2 Expression Targets |
|  | Leptin -> STAT Expression Targets |
|  | Leptin -> ELK/SRF Expression Targets |
|  | Prostaglandin F Expression Targets |
|  | LPA Expression Targets |
|  | PAF/Gq -> AP-1/ATF1/CREB/ERK/SRF Expression Targets |
|  | Morphine Expression Targets |
|  | PAF/Gq -> NF-kB Expression Targets |
|  | S1P Expression Targets |
|  | Proteins/Ions Transporters -> Expression Targets in Lymphoid System and Blood |
|  | Elevated Receptors -> Expression Targets in Lymphoid System and Blood |
|  | F2R/F2RL1 -> Expression Targets in Lymphoid System and Blood |
|  | CD Markers -> Expression Targets in Lymphoid System and Blood |
|  | GPCRs Family -> Expression Targets in Lymphoid System and Blood |
|  | Cell Adhesion Receptors -> Expression Targets in Lymphoid System and Blood |
|  | TNF Receptors -> Expression Targets in Lymphoid System and Blood |
|  | Receptor Tyrosine Kinase Family -> Expression Targets in Lymphoid System and Blood |
|  | Elevated Receptors -> Expression Targets in Bone Marrow |
|  | Interleukin Receptors -> Expression Targets in Lymphoid System and Blood |
|  | C5AR1 -> Expression Targets in Lymphoid System and Blood |
|  | IgE Receptors -> Targets in Lymphoid System and Blood |
|  | Elevated Receptors -> Expression Targets in Thymus |
|  | NGF -> SMAD3/NF-kB Expression Targets |
|  | NGF -> AP-1/TP53/MYC Expression Targets |
|  | NGF -> CREB/CEBPB/MEF2A Expression Targets |
|  | Adenosine Expression Targets |
|  | PDGF -> AP-1/CREB/CREBBP/MYC Expression Targets |
|  | PDGF -> STAT Expression Targets |
|  | NTS Expression Targets |
|  | GAST Expression Targets in GAST Expression Targets |
|  | GNRH2 Expression Targets |
|  | POMC Expression Targets |
|  | EDN1 Expression Targets |
|  | AGT -> CREB Expression Targets |
|  | IFNA1/Gq Expression Targets |
|  | TAC1 Expression Targets |
|  | AGT -> STAT Expression Targets |
|  | PRL/GHR -> NF/kB/ELK/SRF/MYC Expression Targets |
|  | CSF1 -> STAT Expression Targets |
|  | GAS6 -> AP-1/CREB Expression Targets |
|  | KITLG -> STAT Expression Targets |
|  | KITLG -> AP-1/CREB/CREBBP/MYC Expression Targets |
|  | PAF Expression Targets |
|  | CSF1 -> AP-1/CREB/CREBBP/MYC Expression Targets |
|  | PGF -> AP-1/CREB/CREBBP/MYC Expression Targets |
|  | Elevated Receptors -> Expression Targets in Endometrium |
|  | Elevated Receptors -> Expression Targets in Lung |
|  | MERTK Signaling |
|  | TCR -> AP-1 Expression Targets |
|  | TGFA -> STAT Expression Targets |
|  | TGFA -> CTNNB/CTNND Expression Targets |
|  | TGFA/AP-1/ATF Expression Targets |
|  | TGFA -> CREB/CREBBP/ELK-SRF/MYC Expression Targets |
|  | TGFB1-TGFBR2 Expression Targets |
|  | TGFB1-TGFBR1/AP-1 Expression Targets |
|  | TGFB1-ACVRL1 Expression Targets |
|  | FASLG Expression Targets |
|  | TNF -> NF-kB Expression Targets |
|  | TNFSF10 Expression Targets |
|  | TNFSF14 Expression Targets |
|  | TNF -> AP-1 Expression Targets |
|  | CD72 -> AP-1 Expression Targets |
|  | CD40LG -> NF-kB/ELK/SRF -> CREB/NFATC Expression Targets |
|  | TNF -> CREB Expression Targets |
|  | Thrombopoietin -> AP-1/CREB/CREBBP/MYC Expression Targets |
|  | Thrombopoietin -> SP1 Expression Targets |
|  | TLR3 Expression Targets |
|  | TLR4 -> AP-1 Expression Targets |
|  | TLR5 Expression Targets |
|  | TLR7 Expression Targets |
|  | IL1B -> NO Expression Targets |
|  | IL1B -> PGE2 Expression Targets |
|  | TLR1 -> 2/6 Expression Targets |
|  | IL1B Expression Targets |
|  | TLR4 -> NF-kB/IRF Expression Targets |
|  | IL1A Expression Targets |
|  | TLR9 Expression Targets |
|  | ROS in Triggering Vascular Inflammation |
|  | VEGFA -> ATF/CREB/ELK-SRF Expression Targets |
|  | VEGFA -> STAT Expression Targets |
|  | VEGFA -> AP-1/CREBBP/MYC Expression Targets |
|  | VEGFA -> CTNNB/CTNND Expression Targets |
|  | WNT1 Expression Targets |
|  | WNT5A Expression Targets |
|  | WNT Canonical Signaling Expression Targets |
|  | pathway 3 |
|  | pathway 4 |
|  | ace2 |
|  | New Pathway (2) |
|  | PPAR Psoriasis |
|  | pprarg neg, uknown targets, ps-positive |
|  | prarg neg,ukn expres targets, ps-positive |
|  | PPARG negative targets |
|  | pparg expr target\_regulators |
|  | Neighbors of disease exacerbation |
|  | Neighbors of tumor progression |
|  | Neighbors of cancer progression |
|  | Neighbors of inflammatory status |
|  | Neighbors of overall survival |
|  | Neighbors of disease severity |
|  | Neighbors of tumor size |
|  | Neighbors of lesion size |
|  | Neighbors of mortality |
|  | Neighbors of survival rate |
|  | Neighbors of infarct size |
|  | Neighbors of survival time |
|  | Neighbors of insulin sensitivity |
|  | Neighbors of bacterial load |
|  | Neighbors of cancer survival |
|  | Neighbors of therapeutic efficacy |
|  | Neighbors of clinical stage |
|  | Neighbors of lethality |
|  | Neighbors of tumor regression |
|  | Neighbors of recurrence-free survival |
|  | Neighbors of disease-free survival |
|  | Neighbors of tumor response |
|  | Neighbors of macrophage count |
|  | Neighbors of long-term survival |
|  | Neighbors of treatment outcome |
|  | Neighbors of body weight |
|  | Neighbors of body weight gain |
|  | Neighbors of cardiovascular risk |
|  | Neighbors of microvessel density |
|  | Neighbors of tumor recurrence |
|  | Neighbors of tumor promotion |
|  | Neighbors of neutrophil count |
|  | Neighbors of morbidity |
|  | Neighbors of mortality rate |
|  | Neighbors of exhaustion |
|  | Neighbors of adverse outcome |
|  | Neighbors of radioresistance |
|  | Neighbors of cell density |
|  | Neighbors of cancer-specific survival |
|  | Neighbors of progression-free survival |
|  | Neighbors of apoptosis rate |
|  | Neighbors of early mortality |
|  | Neighbors of shear stress |
|  | Neighbors of viral load |
|  | Neighbors of blood pressure |
|  | Neighbors of leukocyte count |
|  | Neighbors of vascular leakage |
|  | Neighbors of body mass index |
|  | Neighbors of vascular density |
|  | Neighbors of muscle mass |
|  | Neighbors of cancer risk |
|  | Neighbors of tumor aggressiveness |
|  | Neighbors of tumor resistance |
|  | Neighbors of median survival |
|  | Neighbors of cancer recurrence |
|  | Neighbors of antimetastatic activity |
|  | Neighbors of radiosensitivity |
|  | Neighbors of breast cancer risk |
|  | Neighbors of proliferation index |
|  | Neighbors of cell permeability |
|  | Neighbors of pregnancy outcome |
|  | Neighbors of gastric cancer risk |
|  | Neighbors of recurrence rate |
|  | Neighbors of pathological response |
|  | Neighbors of lymphocyte count |
|  | Neighbors of monocyte count |
|  | Neighbors of skin thickness |
|  | Neighbors of metabolic response |
|  | Neighbors of comorbidity |
|  | Neighbors of carotid intima-media thickness |
|  | Neighbors of fibroblast phenotype |
|  | Neighbors of disease exacerbation |
|  | Neighbors of tumor progression |
|  | Neighbors of cancer progression |
|  | Neighbors of inflammatory status |
|  | Neighbors of overall survival |
|  | Neighbors of disease severity |
|  | Neighbors of tumor size |
|  | Neighbors of lesion size |
|  | Neighbors of mortality |
|  | Neighbors of survival rate |
|  | Neighbors of infarct size |
|  | Neighbors of survival time |
|  | Neighbors of insulin sensitivity |
|  | Neighbors of bacterial load |
|  | Neighbors of cancer survival |
|  | Neighbors of therapeutic efficacy |
|  | Neighbors of clinical stage |
|  | Neighbors of lethality |
|  | Neighbors of tumor regression |
|  | Neighbors of recurrence-free survival |
|  | Neighbors of disease-free survival |
|  | Neighbors of tumor response |
|  | Neighbors of macrophage count |
|  | Neighbors of long-term survival |
|  | Neighbors of treatment outcome |
|  | Neighbors of body weight |
|  | Neighbors of body weight gain |
|  | Neighbors of cardiovascular risk |
|  | Neighbors of microvessel density |
|  | Neighbors of tumor recurrence |
|  | Neighbors of tumor promotion |
|  | Neighbors of neutrophil count |
|  | Neighbors of morbidity |
|  | Neighbors of mortality rate |
|  | Neighbors of exhaustion |
|  | Neighbors of adverse outcome |
|  | Neighbors of radioresistance |
|  | Neighbors of cell density |
|  | Neighbors of cancer-specific survival |
|  | Neighbors of progression-free survival |
|  | Neighbors of apoptosis rate |
|  | Neighbors of early mortality |
|  | Neighbors of shear stress |
|  | Neighbors of viral load |
|  | Neighbors of blood pressure |
|  | Neighbors of leukocyte count |
|  | Neighbors of vascular leakage |
|  | Neighbors of body mass index |
|  | Neighbors of vascular density |
|  | Neighbors of muscle mass |
|  | Neighbors of cancer risk |
|  | Neighbors of tumor aggressiveness |
|  | Neighbors of tumor resistance |
|  | Neighbors of median survival |
|  | Neighbors of cancer recurrence |
|  | Neighbors of antimetastatic activity |
|  | Neighbors of radiosensitivity |
|  | Neighbors of breast cancer risk |
|  | Neighbors of proliferation index |
|  | Neighbors of cell permeability |
|  | Neighbors of pregnancy outcome |
|  | Neighbors of gastric cancer risk |
|  | Neighbors of recurrence rate |
|  | Neighbors of pathological response |
|  | Neighbors of lymphocyte count |
|  | Neighbors of monocyte count |
|  | Neighbors of skin thickness |
|  | Neighbors of metabolic response |
|  | Neighbors of comorbidity |
|  | Neighbors of carotid intima-media thickness |
|  | Neighbors of fibroblast phenotype |
|  | Neighbors of cell development |
|  | Neighbors of immune response |
|  | Neighbors of cell population |
|  | Neighbors of T-cell development |
|  | Neighbors of inflammatory response |
|  | Neighbors of wound healing |
|  | Neighbors of angiogenesis |
|  | Neighbors of innate immune response |
|  | Neighbors of cell function |
|  | Neighbors of macrophage activation |
|  | Neighbors of cell formation |
|  | Neighbors of adaptive immune response |
|  | Neighbors of osteoclast development |
|  | Neighbors of epithelial to mesenchymal transition |
|  | Neighbors of immunity |
|  | Neighbors of cell proliferative response |
|  | Neighbors of chemotaxis |
|  | Neighbors of dendritic cell differentiation |
|  | Neighbors of cell survival |
|  | Neighbors of osteoclast differentiation |
|  | Neighbors of cell phenotype |
|  | Neighbors of T-cell proliferation |
|  | Neighbors of tissue remodeling |
|  | Neighbors of cell adhesion |
|  | Neighbors of T-cell activation |
|  | Neighbors of endothelial cell proliferation |
|  | Neighbors of fibrogenesis |
|  | Neighbors of tumor growth |
|  | Neighbors of dendritic cell development |
|  | Neighbors of tissue repair |
|  | Neighbors of cell count |
|  | Neighbors of cell infiltration |
|  | Neighbors of cell interaction |
|  | Neighbors of SMC proliferation |
|  | Neighbors of lumen formation |
|  | Neighbors of endothelial cell migration |
|  | Neighbors of adipogenesis |
|  | Neighbors of lipid storage |
|  | Neighbors of cell damage |
|  | Neighbors of pregnancy |
|  | Neighbors of monocyte differentiation |
|  | Neighbors of aging |
|  | Neighbors of cell motility |
|  | Neighbors of bone resorption |
|  | Neighbors of monocyte migration |
|  | Neighbors of fibroblast proliferation |
|  | Neighbors of adipocyte differentiation |
|  | Neighbors of macrophage migration |
|  | Neighbors of microglial activation |
|  | Neighbors of neutrophil recruitment |
|  | Neighbors of regeneration |
|  | Neighbors of cell migration |
|  | Neighbors of cancer growth |
|  | Neighbors of stem cell proliferation |
|  | Neighbors of smooth muscle cell migration |
|  | Neighbors of hepatic regeneration |
|  | Neighbors of transendothelial migration |
|  | Neighbors of phagocytosis |
|  | Neighbors of immune system activation |
|  | Neighbors of leukocyte migration |
|  | Neighbors of leukocyte recruitment |
|  | Neighbors of osteoclast formation |
|  | Neighbors of monocyte recruitment |
|  | Neighbors of cell invasion |
|  | Neighbors of heart function |
|  | Neighbors of hemopoiesis |
|  | Neighbors of bone remodeling |
|  | Neighbors of ossification |
|  | Neighbors of T-cell function |
|  | Neighbors of immune system function |
|  | Neighbors of lung development |
|  | Neighbors of autophagy |
|  | Neighbors of monocyte adhesion |
|  | Neighbors of transcription activation |
|  | Neighbors of senescence |
|  | Neighbors of T-cell response |
|  | Neighbors of tumor immunity |
|  | Neighbors of hepatocyte proliferation |
|  | Neighbors of cell transdifferentiation |
|  | Neighbors of neuroprotection |
|  | Neighbors of stem cell differentiation |
|  | Neighbors of endothelial cell function |
|  | Neighbors of neuronal death |
|  | Neighbors of cell growth |
|  | Neighbors of ROS generation |
|  | Neighbors of epithelial cell proliferation |
|  | Neighbors of first trimester pregnancy |
|  | Neighbors of cancer cell growth |
|  | Neighbors of sensitization |
|  | Neighbors of hemato-encephalic barrier |
|  | Neighbors of cellular immune response |
|  | Neighbors of cellular senescence |
|  | Neighbors of colony formation |
|  | Neighbors of macrophage differentiation |
|  | Neighbors of cell differentiation |
|  | Neighbors of keratinocyte proliferation |
|  | Neighbors of stem cell migration |
|  | Neighbors of life span |
|  | Neighbors of psoriasis |
|  | Neighbors of inflammatory disease |
|  | Neighbors of rheumatoid arthritis |
|  | Neighbors of atherosclerosis |
|  | Neighbors of fibrosis |
|  | Neighbors of colitis |
|  | Neighbors of arthritis |
|  | Neighbors of inflammatory bowel disease |
|  | Neighbors of injury |
|  | Neighbors of atherogenesis |
|  | Neighbors of vascular remodeling |
|  | Neighbors of metastasis |
|  | Neighbors of carcinogenesis |
|  | Neighbors of multiple sclerosis |
|  | Neighbors of chronic inflammation |
|  | Neighbors of inflammation |
|  | Neighbors of tumor microenvironment |
|  | Neighbors of pathological angiogenesis |
|  | Neighbors of obesity |
|  | Neighbors of vascular disease |
|  | Neighbors of diabetes mellitus |
|  | Neighbors of asthma |
|  | Neighbors of pneumonia |
|  | Neighbors of hyperplasia |
|  | Neighbors of osteoarthritis |
|  | Neighbors of liver fibrosis |
|  | Neighbors of chronic obstructive pulmonary disease |
|  | Neighbors of myocardial infarction |
|  | Neighbors of leukocyte infiltration |
|  | Neighbors of reperfusion injury |
|  | Neighbors of airway inflammation |
|  | Neighbors of hepatitis |
|  | Neighbors of insulin resistance |
|  | Neighbors of infection |
|  | Neighbors of vascular injury |
|  | Neighbors of renal injury |
|  | Neighbors of preeclampsia |
|  | Neighbors of macrophage infiltration |
|  | Neighbors of liver injury |
|  | Neighbors of neuroinflammation |
|  | Neighbors of sepsis |
|  | Neighbors of death |
|  | Neighbors of type 1 diabetes |
|  | Neighbors of autoimmune disease |
|  | Neighbors of ischemic stroke |
|  | Neighbors of dermatitis |
|  | Neighbors of neutrophil infiltration |
|  | Neighbors of heart disease |
|  | Neighbors of diabetic nephropathy |
|  | Neighbors of lung injury |
|  | Neighbors of immunopathology |
|  | Neighbors of systemic sclerosis |
|  | Neighbors of hepatocellular carcinoma |
|  | Neighbors of autoimmunity |
|  | Neighbors of acute lung injury |
|  | Neighbors of gastric cancer |
|  | Neighbors of atherosclerotic plaque |
|  | Neighbors of colorectal cancer |
|  | Neighbors of cardiac remodeling |
|  | Neighbors of breast cancer |
|  | Neighbors of ischemia |
|  | Neighbors of systemic lupus erythematosus |
|  | Neighbors of melanoma |
|  | Neighbors of cartilage degeneration |
|  | Neighbors of interstitial fibrosis |
|  | Neighbors of neoplasm invasion |
|  | Neighbors of kidney disease |
|  | Neighbors of heart failure |
|  | Neighbors of lung disease |
|  | Neighbors of pulmonary fibrosis |
|  | Neighbors of cardiovascular disease |
|  | Neighbors of stroke |
|  | Neighbors of cancer |
|  | Neighbors of cerebral ischemia |
|  | Neighbors of type 2 diabetes |
|  | Neighbors of neoplasm |
|  | Neighbors of endometriosis |
|  | Neighbors of vasculitis |
|  | Neighbors of atrophy |
|  | Neighbors of respiratory hypersensitivity |
|  | Neighbors of synovitis |
|  | Neighbors of coronary artery disease |
|  | Neighbors of rupture |
|  | Neighbors of endothelial cell dysfunction |
|  | Neighbors of liver disease |
|  | Neighbors of prostate cancer |
|  | Neighbors of hypertrophy |
|  | Neighbors of osteolysis |
|  | Neighbors of ovarian cancer |
|  | Neighbors of pancreatic cancer |
|  | Neighbors of hypertension |
|  | Neighbors of intimal hyperplasia |
|  | Neighbors of allograft rejection |
|  | Neighbors of proteinuria |
|  | Neighbors of epithelial cell |
|  | Neighbors of keratinocyte |
|  | Neighbors of vascular smooth muscle cell |
|  | Neighbors of umbilical vein endothelial cell |
|  | Neighbors of monocyte |
|  | Neighbors of endometrial stromal cell |
|  | Neighbors of alveolar macrophage |
|  | Neighbors of synoviocyte |
|  | Neighbors of macrophage |
|  | Neighbors of adipocyte |
|  | Neighbors of activated monocyte |
|  | Neighbors of fibroblast |
|  | Neighbors of airway epithelial cell |
|  | Neighbors of PBMC |
|  | Neighbors of stromal cell |
|  | Neighbors of airway smooth muscle cell |
|  | Neighbors of osteoblast |
|  | Neighbors of pneumocyte |
|  | Neighbors of endometrium cell |
|  | Neighbors of microglia |
|  | Neighbors of trophoblast |
|  | Neighbors of intestine epithelium cell |
|  | Neighbors of mononuclear cell |
|  | Neighbors of hepatic stellate cell |
|  | Neighbors of hepatocyte |
|  | Neighbors of decidual cell |
|  | Neighbors of kidney mesangial cell |
|  | Neighbors of endothelial cell |
|  | Neighbors of dendritic cell |
|  | Neighbors of Kuppfer cell |
|  | Neighbors of proadipocyte |
|  | Neighbors of kidney tubule cell |
|  | Neighbors of inflammatory cell |
|  | Neighbors of bone marrow macrophage |
|  | Neighbors of peritoneal macrophage |
|  | Neighbors of osteoclast |
|  | Neighbors of neutrophil |
|  | Neighbors of smooth muscle myocyte |
|  | Neighbors of astrocyte |
|  | Neighbors of bone marrow derived dendritic cell |
|  | Neighbors of lung fibroblast |
|  | Neighbors of activated T-cell |
|  | Neighbors of mesothelial cell |
|  | Neighbors of mononuclear phagocyte |
|  | Neighbors of capillary endothelial cell |
|  | Neighbors of immunocompetent cell |
|  | Neighbors of granulocyte |
|  | Neighbors of cardiac myocyte |
|  | Neighbors of cancer-associated fibroblast |
|  | Neighbors of myeloid cell |
|  | Neighbors of mesenchymal stroma cell |
|  | Neighbors of corneal epithelial cell |
|  | Neighbors of cytotrophoblast |
|  | Neighbors of stem cell |
|  | Neighbors of tumor infiltrating macrophage |
|  | Neighbors of sinusoidal cell |
|  | Neighbors of B-cell |
|  | Neighbors of granulosa cell |
|  | Neighbors of leukocyte |
|  | Neighbors of chondrocyte |
|  | Neighbors of insulin-secreting cell |
|  | Neighbors of mesenchymal cell |
|  | Neighbors of T-cell |
|  | Neighbors of M1 macrophage |
|  | Neighbors of myofibroblast |
|  | Neighbors of pancreatic stellate cell |
|  | Neighbors of mast cell |
|  | Neighbors of bone marrow stromal cell |
|  | Neighbors of spleen cell |
|  | Neighbors of brain capillary endothelial cell |
|  | Neighbors of neuroglia |
|  | Neighbors of thyroid epithelial cell |
|  | Neighbors of aortic smooth muscle cell |
|  | Neighbors of hematopoietic cell |
|  | Neighbors of adipose macrophage |
|  | Neighbors of podocyte |
|  | Neighbors of mammary epithelial cell |
|  | Neighbors of conventional dendritic cell |
|  | Neighbors of pericyte |
|  | Neighbors of antigen-presenting cell |
|  | Neighbors of epidermis cell |
|  | Neighbors of phagocyte |
|  | Neighbors of kidney cell |
|  | Neighbors of syncytiotrophoblast |
|  | Neighbors of lymphocyte |
|  | Neighbors of Langerhans cell |
|  | Neighbors of decidual natural killer cell |
|  | Neighbors of thymic epithelial cell |
|  | Neighbors of brain cell |
|  | Neighbors of M2 macrophage |
|  | Neighbors of peritoneum cell |
|  | Neighbors of lymphoid cell |
|  | Neighbors of retina cell |
|  | Neighbors of blood cell |
|  | Neighbors of skin cell |
|  | Neighbors of peripheral lymphocyte |
|  | Neighbors of activated B-cell |
|  | Neighbors of lung |
|  | Neighbors of liver |
|  | Neighbors of kidney |
|  | Neighbors of skin |
|  | Neighbors of intestine |
|  | Neighbors of lymph node |
|  | Neighbors of colon |
|  | Neighbors of airway |
|  | Neighbors of blood vessel |
|  | Neighbors of heart |
|  | Neighbors of aorta |
|  | Neighbors of gastrointestinal tract |
|  | Neighbors of microvessel |
|  | Neighbors of bone |
|  | Neighbors of placenta |
|  | Neighbors of synovial membrane |
|  | Neighbors of uterus |
|  | Neighbors of esophagus |
|  | Neighbors of periodontium |
|  | Neighbors of retina |
|  | Neighbors of brain |
|  | Neighbors of ovary |
|  | Neighbors of breast |
|  | Neighbors of blood vessel wall |
|  | Neighbors of stomach |
|  | Neighbors of islets of Langerhans |
|  | Neighbors of joint |
|  | Neighbors of artery |
|  | Neighbors of pancreas |
|  | Neighbors of prostate |
|  | Neighbors of central nervous system |
|  | Neighbors of kidney glomerulus |
|  | Neighbors of colorectal region |
|  | Neighbors of decidua |
|  | Neighbors of amnion |
|  | Neighbors of immune system |
|  | Neighbors of spleen |
|  | Neighbors of peritoneum |
|  | Neighbors of allantois |
|  | Neighbors of alveolus |
|  | Neighbors of uterine cervix |
|  | Neighbors of omentum |
|  | Neighbors of vein |
|  | Neighbors of villus |
|  | Neighbors of bronchus |
|  | Neighbors of glands |
|  | Neighbors of nose |
|  | Neighbors of eye |
|  | Neighbors of cornea |
|  | Neighbors of hippocampus |
|  | Neighbors of intervertebral disc |
|  | Neighbors of mammary gland |
|  | Neighbors of vagina |
|  | Neighbors of vitreous body |
|  | Neighbors of myelin sheath |
|  | Neighbors of bladder |
|  | Neighbors of retina blood vessel |
|  | Neighbors of nasopharynx |
|  | Neighbors of periodontal ligament |
|  | Neighbors of salivary gland |
|  | Neighbors of trachea |
|  | Neighbors of corpus luteum of ovary |
|  | Neighbors of nerve |
|  | Neighbors of endocrine system |
|  | Neighbors of small intestine |
|  | Neighbors of amniotic cavity |
|  | Neighbors of knee joint |
|  | Neighbors of hypothalamus |
|  | Neighbors of NF-kB family |
|  | Neighbors of oxidized LDL |
|  | Neighbors of JNK |
|  | Neighbors of IL1 family |
|  | Neighbors of PPAR |
|  | Neighbors of Jun/Fos |
|  | Neighbors of NFKBI |
|  | Neighbors of PKC |
|  | Neighbors of PI3K |
|  | Neighbors of STAT family |
|  | Neighbors of mitogen-activated protein kinase |
|  | Neighbors of IFNAR ligand |
|  | Neighbors of transforming growth factor |
|  | Neighbors of TLR |
|  | Neighbors of cytokine |
|  | Neighbors of interferon |
|  | Neighbors of interleukin |
|  | Neighbors of JAK |
|  | Neighbors of MEK1/2 |
|  | Neighbors of PDGF |
|  | Neighbors of Ras GTPase |
|  | Neighbors of ERK1/2 |
|  | Neighbors of growth factor |
|  | Neighbors of PKA |
|  | Neighbors of LDL |
|  | Neighbors of NF-AT family |
|  | Neighbors of beta adrenoceptor |
|  | Neighbors of chorionic gonadotropin |
|  | Neighbors of IKBK |
|  | Neighbors of adipokine |
|  | Neighbors of protein tyrosine kinase |
|  | Neighbors of NAD(P)H oxidase |
|  | Neighbors of IgG |
|  | Neighbors of SMAD subfamily |
|  | Neighbors of AMPK |
|  | Neighbors of LXR |
|  | Neighbors of HDL |
|  | Neighbors of p38 |
|  | Neighbors of inflammatory cytokine |
|  | Neighbors of IL23 |
|  | Neighbors of estrogen receptor |
|  | Neighbors of T-cell receptor |
|  | Neighbors of histone deacetylase |
|  | Neighbors of PDGF-BB |
|  | Neighbors of IL1R |
|  | Neighbors of HIF-1 |
|  | Neighbors of GPCR |
|  | Neighbors of IL12 |
|  | Neighbors of Notch |
|  | Neighbors of prostaglandin-endoperoxide synthase |
|  | Neighbors of heat shock protein 90 |
|  | Neighbors of lectin |
|  | Neighbors of fibrinogen |
|  | Neighbors of aldehyde reductase |
|  | Neighbors of mitogen-activated protein kinase kinase |
|  | Neighbors of interleukin-35 |
|  | Neighbors of TSH |
|  | Neighbors of endothelin |
|  | Neighbors of NOS |
|  | Neighbors of superoxide dismutase |
|  | Neighbors of proteasome endopeptidase complex |
|  | Neighbors of class nuclear receptor with C4 zinc fingers |
|  | Neighbors of non-coding RNA |
|  | Neighbors of histone H3 |
|  | Neighbors of STAT5 |
|  | Neighbors of alpha2 adrenoceptor |
|  | Neighbors of vitronectin receptor (integrin) |
|  | Neighbors of PP2A |
|  | Neighbors of retinoid-X receptor subfamily |
|  | Neighbors of SAPK |
|  | Neighbors of WNT |
|  | Neighbors of gonadotropins |
|  | Neighbors of immunoglobulin |
|  | Neighbors of PP2B |
|  | Neighbors of phospholipase |
|  | Neighbors of AHR subfamily |
|  | Neighbors of ITG |
|  | Neighbors of endostatins |
|  | Neighbors of CD3 complex |
|  | Neighbors of relaxin |
|  | Neighbors of TLR1/2 heterodimer |
|  | Neighbors of TORC1 |
|  | Neighbors of angiotensin receptor |
|  | Neighbors of RA receptor |
|  | Neighbors of histone |
|  | Neighbors of Rho kinase |
|  | Neighbors of neuropeptides |
|  | Neighbors of pancreatic elastase |
|  | Neighbors of histone acetyltransferase |
|  | Neighbors of phosphodiesterase |
|  | Neighbors of caspase |
|  | Neighbors of phosphoinositide phospholipase C |
|  | Neighbors of deacetylase |
|  | Neighbors of TLR4/Myd88 |
|  | comon ps\_pos, pprarg\_neg targets |
|  | Model of PPARG signaling in psoriasis |
|  | PPARG negative regulators and targets |
|  | Model of PPARG related pathways in psoriasis (short version) |
|  | sars-cov no binding |
|  | Overview. Diseases of the respiratory (chapter 9) |
|  | Overview. Endocrine and metabolic diseases (chapter 4) |
|  | 1\_Inflammation in keratinocytes in the pathology of acne |
|  | 2\_Acute phase initiation in atopic dermatitis |
|  | 2\_Interleukin-17 and interleukin-22 signaling in psoriasis |
|  | 1\_The role of Toll-like receptors in pyelonephritis |
|  | 2\_The role of endothelial cells in pyelonephritis |
|  | 3\_Endoplasmic reticulum stress in age-related macular degeneration |
|  | 1\_Insufficient activation of immune response in the middle ear epithelium cells in otitis media |
|  | 1\_Cigarette smoke causes dysfunction and production of inflammatory mediators in alveolar epithelial cells |
|  | 3\_Role of alveolar macrophages in pulmonary tissue destruction in COPD |
|  | 2\_3\_Eosinophilia and neutrophilia in asthma: Neutrophil chemotaxis and activation |
|  | 2\_2\_Eosinophilia and neutrophilia in asthma: eosinophil apoptosis |
|  | 2\_Paneth cell dysfunction in Crohn's disease |
|  | 4\_2\_Genes with mutations currently associated with ulcerative colitis |
|  | 3\_Leukocyte migration toward the endothelial cells in the intestine microvasculature |
|  | 4\_1\_Polymorphisms associated with inflammatory bowel diseases |
|  | 5\_Human papilloma virus E6 and E7 proteins promote epithelial cell proliferation |
|  | 1\_SERPINA1-associated pulmonary emphysema |
|  | 2\_SERPINA1-associated liver damage |
|  | drug-target pairs direct regulation |
|  | drug-target moltransport |
|  | Interleukin-17 and interleukin-22 signaling in psoriasis |

---

|  |  |
| --- | --- |
| Group | Genes Associated with Systemic Lupus Erythematosus |
|  | Genes with Mutations Associated with Cystic Fibrosis |
|  | Genes with Mutations Associated with Systemic Scleroderma |
|  | Chemokines, Growth Factors and Receptors Involved in Cancer Cells Chemotaxis |
|  | other partners for SARS |

---

|  |  |
| --- | --- |
| Source | curated |
|  | GO component |

---

|  |  |
| --- | --- |
| MedScan ID | 3576 |

---

|  |  |
| --- | --- |
| LocusLink ID | 3576 |
|  | 280828 |
|  | 567083 |

---

|  |  |
| --- | --- |
| Alias | C-X-C motif chemokine ligand 8 |
|  | IL8\_HUMAN |
|  | LYNAP |
|  | lymphocyte derived neutrophil activating peptide |
|  | monocyte-derived neutrophil-activating peptide |
|  | LECT |
|  | Granulocyte chemotactic protein 1 |
|  | neutrophil-activating peptide I |
|  | IL8 |
|  | CXC chemokine ligand 8 |
|  | interleukin IL VIIIs |
|  | interleukin IL8 |
|  | chemokine CXC ligand 8 |
|  | CXCL8 |
|  | small inducible cytokine subfamily B, member 8 |
|  | IL VIII |
|  | small inducible cytokine subfamily B, member VIII |
|  | Lymphocyte-derived neutrophil- activating factor |
|  | Neutrophil-activating factor |
|  | polymorphonuclear granulocyte activating factor |
|  | Granulocyte chemotactic protein I |
|  | IL VIIIs |
|  | interleukin VIII |
|  | b-ENAP |
|  | CXC chemokine ligand VIII |
|  | GCP-1 |
|  | monocyte-derived neutrophil chemotactic factor |
|  | neutrophil activating peptide |
|  | Neutrophil-activating protein I |
|  | GCP I |
|  | chemokine CXCL8 |
|  | C-X-C motif chemokine 8 |
|  | beta-thromboglobulin-like protein |
|  | neutrophil-activating peptide 1 |
|  | beta endothelial cell-derived neutrophil activating peptide |
|  | granulocyte chemotactic peptide |
|  | LUCT |
|  | MONAP |
|  | lung giant cell carcinoma-derived chemotactic protein |
|  | LUCT/interleukin-8 |
|  | interleukin 8 |
|  | interleukin IL VIII |
|  | alveolar macrophage chemotactic factor I |
|  | neutrophil attracting peptide |
|  | monocyte derived neutrophil-activating protein |
|  | OTTHUMP00000199825 |
|  | interleukin IL8\_HUMAN |
|  | MDNCF |
|  | T-cell chemotactic factor |
|  | CXCL8 chemokine |
|  | AMCF-I |
|  | chemokine (C-X-C motif) ligand 8 |
|  | LOC133094 |
|  | SCYB8 |
|  | Neutrophil-activating protein 1 |
|  | 3-10C |
|  | NAF |
|  | GCP1 |
|  | NAP1 |
|  | NAP-1 |
|  | emoctakin |
|  | tumor necrosis factor-induced gene 1 |
|  | interleukin-8 |
|  | IL[8] |
|  | T cell chemotactic factor |
|  | lymphocyte-derived neutrophil-activating factor |
|  | Interleukin-IL8 |
|  | Neutrophil- activating protein 1 |
|  | Neutrophil-activating factor |
|  | Neutrophil- activating protein I |
|  | K60 |
|  | TSG-1 |
|  | Monocyte-derived neutrophil chemotactic factor |
|  | TSG I |
|  | K-60 |
|  | IL-8 |
|  | protein 3-10C |
|  | chemokine (c-x-c motif) ligand VIII |
|  | K(60) |
|  | LOC567083 |
|  | chromosome |
|  | scaffold |
|  | similar to interleukin 8 |

---

|  |  |
| --- | --- |
| GO ID | 0008009 |
|  | 0005153 |
|  | 0007186 |
|  | 0036499 |
|  | 0001525 |
|  | 0061844 |
|  | 0019722 |
|  | 0007050 |
|  | 0044344 |
|  | 0071347 |
|  | 0071222 |
|  | 0071356 |
|  | 0070098 |
|  | 0006935 |
|  | 0019221 |
|  | 0048566 |
|  | 0006955 |
|  | 0050930 |
|  | 0006954 |
|  | 0035556 |
|  | 0030595 |
|  | 0045744 |
|  | 0008285 |
|  | 0042119 |
|  | 0030593 |
|  | 0045766 |
|  | 0090023 |
|  | 0031623 |
|  | 0030155 |
|  | 2000535 |
|  | 0045091 |
|  | 0034976 |
|  | 0002237 |
|  | 0007165 |
|  | 0005576 |
|  | 0005615 |
|  | 0006928 |
|  | 0005622 |
|  | 0006987 |
|  | 0044267 |
|  | 0030968 |
|  | 0005515 |
|  | 0007267 |
|  | 0007242 |
|  | 0023034 |
|  | 0007600 |

---

|  |  |
| --- | --- |
| KEGG ID | hsa:3576 |

---

|  |  |
| --- | --- |
| MedLine Reference | 10343098 |
|  | 10368283 |
|  | 10376933 |
|  | 10491366 |
|  | 10620615 |
|  | 10707023 |
|  | 10734056 |
|  | 10775602 |
|  | 10820279 |
|  | 10823949 |
|  | 10880251 |
|  | 10881932 |
|  | 10929056 |
|  | 10982368 |
|  | 10985244 |
|  | 10985253 |
|  | 11023497 |
|  | 11069235 |
|  | 11083887 |
|  | 11160671 |
|  | 11328384 |
|  | 11483765 |
|  | 11564821 |
|  | 11756416 |
|  | 11784713 |
|  | 11814358 |
|  | 11854210 |
|  | 11855786 |
|  | 11868823 |
|  | 11876744 |
|  | 11877327 |
|  | 11884401 |
|  | 11884459 |
|  | 11888846 |
|  | 11896938 |
|  | 11910304 |
|  | 11911801 |
|  | 11950481 |
|  | 11956621 |
|  | 11960393 |
|  | 11964077 |
|  | 11966578 |
|  | 11973337 |
|  | 11989790 |
|  | 11999552 |
|  | 12027404 |
|  | 12033786 |
|  | 12034575 |
|  | 12039947 |
|  | 12055238 |
|  | 12055326 |
|  | 12056817 |
|  | 12060853 |
|  | 12090473 |
|  | 12091490 |
|  | 12093676 |
|  | 12096924 |
|  | 12096927 |
|  | 12100025 |
|  | 12101072 |
|  | 12115500 |
|  | 12117737 |
|  | 12117926 |
|  | 12117969 |
|  | 12117995 |
|  | 12123753 |
|  | 12126643 |
|  | 12133438 |
|  | 12139952 |
|  | 12149127 |
|  | 12150710 |
|  | 12151316 |
|  | 12151344 |
|  | 12163591 |
|  | 12165085 |
|  | 12169092 |
|  | 12169272 |
|  | 12186702 |
|  | 12186913 |
|  | 12187073 |
|  | 12195386 |
|  | 12223528 |
|  | 12225945 |
|  | 12239630 |
|  | 12359770 |
|  | 12364441 |
|  | 12366401 |
|  | 12382118 |
|  | 12384994 |
|  | 12388718 |
|  | 12388959 |
|  | 12391099 |
|  | 12393171 |
|  | 12410798 |
|  | 12453441 |
|  | 12477932 |
|  | 12480084 |
|  | 12485855 |
|  | 12496258 |
|  | 12502445 |
|  | 12513912 |
|  | 12520365 |
|  | 12524080 |
|  | 12529421 |
|  | 12530093 |
|  | 12533683 |
|  | 12543081 |
|  | 12547728 |
|  | 12548717 |
|  | 12565901 |
|  | 12573991 |
|  | 12576442 |
|  | 12584113 |
|  | 12586630 |
|  | 12594001 |
|  | 12594058 |
|  | 12615831 |
|  | 12626597 |
|  | 12628493 |
|  | 12632066 |
|  | 12634636 |
|  | 12637525 |
|  | 12646250 |
|  | 12653788 |
|  | 12654635 |
|  | 12654834 |
|  | 12660426 |
|  | 12662377 |
|  | 12702497 |
|  | 12707271 |
|  | 12720537 |
|  | 12727029 |
|  | 12729795 |
|  | 12738388 |
|  | 12744776 |
|  | 12748056 |
|  | 12748173 |
|  | 12777373 |
|  | 12782302 |
|  | 12792476 |
|  | 12797546 |
|  | 12802400 |
|  | 1281554 |
|  | 12818188 |
|  | 12820969 |
|  | 12839933 |
|  | 12839942 |
|  | 12849706 |
|  | 12850808 |
|  | 12855659 |
|  | 12857601 |
|  | 12871882 |
|  | 12876073 |
|  | 12898436 |
|  | 12898448 |
|  | 12901801 |
|  | 12902511 |
|  | 12909591 |
|  | 12919697 |
|  | 12933891 |
|  | 12952969 |
|  | 12960242 |
|  | 13129857 |
|  | 1391327 |
|  | 14500465 |
|  | 14500470 |
|  | 14500479 |
|  | 14530367 |
|  | 14550286 |
|  | 14578856 |
|  | 14623984 |
|  | 14625302 |
|  | 14630611 |
|  | 14634582 |
|  | 14634584 |
|  | 14641180 |
|  | 14645117 |
|  | 14657869 |
|  | 14669337 |
|  | 14672330 |
|  | 14676125 |
|  | 14688120 |
|  | 14714557 |
|  | 14718619 |
|  | 14725568 |
|  | 14729508 |
|  | 14730209 |
|  | 14756945 |
|  | 14767470 |
|  | 14977935 |
|  | 15001458 |
|  | 15021975 |
|  | 15039334 |
|  | 15047831 |
|  | 15057743 |
|  | 15063730 |
|  | 15069060 |
|  | 15072962 |
|  | 15077296 |
|  | 15079071 |
|  | 15085176 |
|  | 15096327 |
|  | 15102084 |
|  | 15120188 |
|  | 15143062 |
|  | 15146413 |
|  | 15152368 |
|  | 15158775 |
|  | 15169673 |
|  | 15178568 |
|  | 15178703 |
|  | 15181190 |
|  | 15194816 |
|  | 15196572 |
|  | 15203561 |
|  | 15203565 |
|  | 15208657 |
|  | 15208668 |
|  | 15209388 |
|  | 15214047 |
|  | 15218164 |
|  | 15222686 |
|  | 15271933 |
|  | 15273081 |
|  | 15280021 |
|  | 15280372 |
|  | 15292066 |
|  | 15297389 |
|  | 15300588 |
|  | 15302608 |
|  | 15321994 |
|  | 15321998 |
|  | 15331599 |
|  | 15337758 |
|  | 15342208 |
|  | 15356171 |
|  | 15356575 |
|  | 15358661 |
|  | 15364108 |
|  | 15379213 |
|  | 15385484 |
|  | 15453805 |
|  | 15454487 |
|  | 15469436 |
|  | 15489227 |
|  | 15496610 |
|  | 15498828 |
|  | 15514971 |
|  | 15521010 |
|  | 15528384 |
|  | 15529362 |
|  | 15531763 |
|  | 15534087 |
|  | 15541716 |
|  | 15542429 |
|  | 15556942 |
|  | 15564333 |
|  | 15569594 |
|  | 15579374 |
|  | 15579377 |
|  | 15583145 |
|  | 15585560 |
|  | 15585673 |
|  | 15591054 |
|  | 15607204 |
|  | 15607368 |
|  | 15609321 |
|  | 15613411 |
|  | 15615716 |
|  | 15630185 |
|  | 15641067 |
|  | 15650393 |
|  | 15650394 |
|  | 15652404 |
|  | 15652492 |
|  | 15659384 |
|  | 15661856 |
|  | 15664665 |
|  | 15667946 |
|  | 15679580 |
|  | 15701651 |
|  | 15731050 |
|  | 15746434 |
|  | 15760459 |
|  | 15760676 |
|  | 15784717 |
|  | 15803062 |
|  | 15805227 |
|  | 15828019 |
|  | 15831558 |
|  | 15845475 |
|  | 15845476 |
|  | 15845864 |
|  | 15862344 |
|  | 15866212 |
|  | 15894298 |
|  | 15897801 |
|  | 15902922 |
|  | 15914533 |
|  | 15945132 |
|  | 15962221 |
|  | 15972644 |
|  | 15976103 |
|  | 15976326 |
|  | 15979992 |
|  | 15987860 |
|  | 16004996 |
|  | 16029496 |
|  | 16038881 |
|  | 16039610 |
|  | 16052590 |
|  | 16085794 |
|  | 16088182 |
|  | 16096323 |
|  | 16098041 |
|  | 16098254 |
|  | 16101867 |
|  | 16107255 |
|  | 16125276 |
|  | 16134994 |
|  | 16164755 |
|  | 16166417 |
|  | 16168518 |
|  | 16185356 |
|  | 16195477 |
|  | 16195815 |
|  | 16197369 |
|  | 16204411 |
|  | 16220540 |
|  | 16240219 |
|  | 16251188 |
|  | 16251427 |
|  | 16270354 |
|  | 16280123 |
|  | 16289597 |
|  | 16293628 |
|  | 16293707 |
|  | 16299055 |
|  | 16322472 |
|  | 16365152 |
|  | 16373669 |
|  | 16387844 |
|  | 16394009 |
|  | 16406804 |
|  | 16491014 |
|  | 16498082 |
|  | 16499908 |
|  | 16525644 |
|  | 16541418 |
|  | 16545602 |
|  | 16547413 |
|  | 16552338 |
|  | 16552751 |
|  | 16554734 |
|  | 16581825 |
|  | 16632517 |
|  | 16679918 |
|  | 16687414 |
|  | 1673289 |
|  | 16757060 |
|  | 16769764 |
|  | 16777061 |
|  | 16793206 |
|  | 16794530 |
|  | 16795034 |
|  | 16797773 |
|  | 16820307 |
|  | 16824064 |
|  | 16825597 |
|  | 16846747 |
|  | 16864907 |
|  | 16864908 |
|  | 16873245 |
|  | 16890267 |
|  | 16926411 |
|  | 16937534 |
|  | 16939660 |
|  | 16973972 |
|  | 16990258 |
|  | 17035306 |
|  | 17078003 |
|  | 17115422 |
|  | 1840701 |
|  | 1895676 |
|  | 1988949 |
|  | 2007144 |
|  | 2184886 |
|  | 2648569 |
|  | 2663993 |
|  | 2664463 |
|  | 2681204 |
|  | 2953813 |
|  | 3223947 |
|  | 3260265 |
|  | 7673721 |
|  | 7695626 |
|  | 8077676 |
|  | 8081002 |
|  | 8381837 |
|  | 8626516 |
|  | 8662698 |
|  | 8663179 |
|  | 8702798 |
|  | 9141135 |
|  | 9323208 |
|  | 9363947 |
|  | 9378998 |
|  | 9464567 |
|  | 9510190 |
|  | 9551928 |
|  | 9659156 |
|  | 9828130 |
|  | 9921412 |
|  | 10361232 |
|  | 11978786 |
|  | 14711052 |
|  | 15489334 |
|  | 1639201 |
|  | 16547804 |
|  | 16620395 |
|  | 16927373 |
|  | 16928771 |
|  | 16978071 |
|  | 16996240 |
|  | 17008880 |
|  | 17020755 |
|  | 17020928 |
|  | 17022986 |
|  | 17024463 |
|  | 17061983 |
|  | 17075576 |
|  | 17141217 |
|  | 17174061 |
|  | 17189320 |
|  | 17202379 |
|  | 17203209 |
|  | 17204468 |
|  | 17207890 |
|  | 17211588 |
|  | 17215524 |
|  | 17220369 |
|  | 17240051 |
|  | 17244794 |
|  | 17257314 |
|  | 17268170 |
|  | 17277102 |
|  | 17304101 |
|  | 17307163 |
|  | 17309748 |
|  | 17356794 |
|  | 17420005 |
|  | 17433258 |
|  | 17458901 |
|  | 17466952 |
|  | 17494793 |
|  | 17498253 |
|  | 17498967 |
|  | 17513455 |
|  | 17538964 |
|  | 17540779 |
|  | 17543146 |
|  | 17550933 |
|  | 17565687 |
|  | 17592301 |
|  | 17620090 |
|  | 17638896 |
|  | 2005614 |
|  | 2145175 |
|  | 2170450 |
|  | 2182630 |
|  | 2200751 |
|  | 2212672 |
|  | 2456256 |
|  | 2523801 |
|  | 2555418 |
|  | 2648135 |
|  | 2655583 |
|  | 2659722 |
|  | 3057503 |
|  | 3279957 |
|  | 3322281 |
|  | 3480540 |
|  | 8631339 |
|  | 11252171 |
|  | 11768129 |
|  | 12016516 |
|  | 15372997 |
|  | 8737490 |
|  | 9922392 |

---

|  |  |
| --- | --- |
| Organism | Homo sapiens {Organism urn:agi-taxid:9606} |
|  | Homo sapiens |
|  | Bos taurus |
|  | Danio rerio |

---

|  |  |
| --- | --- |
| OMIM ID | 146930 |

---

|  |  |
| --- | --- |
| Hugo ID | 6025 |
|  | HGNC:6025 |

---

|  |  |
| --- | --- |
| Human chromosome position | 4q13.3 |
|  | 4q13-q21 |

---

|  |  |
| --- | --- |
| Swiss-Prot Accession | A0A024RDA5 |
|  | P10145 |
|  | P10145.1 |
|  | B2R4L8 |
|  | Q6FGF6 |
|  | Q6LAE6 |
|  | Q96RG6 |
|  | Q9C077 |
|  | Q9UCE1 |
|  | Q9UCR8 |
|  | Q9UCR9 |
|  | Q9UCS0 |
|  | AAB21773 |
|  | AAB23944 |
|  | Q6LDS6 |
|  | IL8\_HUMAN |

---

|  |  |
| --- | --- |
| PIR ID | A37034 |

---

|  |  |
| --- | --- |
| GenBank ID | NC\_000004 |
|  | NM\_000584 |
|  | NP\_000575 |
|  | NM\_001354840 |
|  | NP\_001341769 |
|  | NG\_029889 |
|  | AC112518 |
|  | AF385628 |
|  | AAK60276 |
|  | CH471057 |
|  | EAX05687 |
|  | EAX05688 |
|  | EAX05689 |
|  | D14283 |
|  | BAA03245 |
|  | HC462818 |
|  | CBK51144 |
|  | HI519526 |
|  | CBX54344 |
|  | M28130 |
|  | AAA59158 |
|  | AJ227913 |
|  | AK131067 |
|  | AK311874 |
|  | BAG34815 |
|  | BC013615 |
|  | AAH13615 |
|  | BG497712 |
|  | BT007067 |
|  | AAP35730 |
|  | CR542151 |
|  | CAG46948 |
|  | DA671239 |
|  | M17017 |
|  | AAA35611 |
|  | M26383 |
|  | AAA36323 |
|  | Y00787 |
|  | CAA68742 |
|  | Z11686 |
|  | CAA77745 |
|  | P10145 |
|  | NC\_018915 |
|  | AMYH02009469 |
|  | DQ890564 |
|  | ABM92181 |
|  | DQ893727 |
|  | ABM84653 |
|  | M23344 |
|  | AAA74722 |
|  | AC\_000136 |
|  | ABBA01022993 |
|  | NT\_022778 |
|  | NW\_001838914 |
|  | NW\_922162 |
|  | AC\_000047 |
|  | CR594973 |
|  | CR595357 |
|  | CR600500 |
|  | CR601533 |
|  | CR601902 |
|  | CR603686 |
|  | CR619554 |
|  | CR623683 |
|  | CR623827 |
|  | NT\_006216 |
|  | M20922 |
|  | AAA66353 |
|  | X87888 |
|  | CAA61140 |
|  | AAB21773 |
|  | AAB23944 |
|  | Q6LDS6 |
|  | AAA63206 |
|  | AF123369 |
|  | CAA54789 |
|  | AAA52849 |
|  | M31608 |
|  | BAC01669 |
|  | AB064041 |
|  | X77737 |
|  | M62731 |
|  | AAM75835 |
|  | 119626093 |
|  | 119626094 |
|  | 123994103 |
|  | 124359 |
|  | 14326461 |
|  | 15488984 |
|  | 179580 |
|  | 183043 |
|  | 185248 |
|  | 185490 |
|  | 186368 |
|  | 188628 |
|  | 21669289 |
|  | 21702298 |
|  | 219916 |
|  | 247142 |
|  | 258901 |
|  | 30582973 |
|  | 33959 |
|  | 34519 |
|  | 49457298 |
|  | 535097 |
|  | 535769 |
|  | 74724764 |
|  | 871269 |
|  | 119626092 |
|  | 124126817 |
|  | NT\_086651 |
|  | 1699354 |
|  | 2829675 |
|  | 56405844 |
|  | 56549077 |
|  | 7158874 |
|  | 73587443 |
|  | 75053328 |
|  | AAB37483 |
|  | AAD02808 |
|  | AAF37575 |
|  | AAI03311 |
|  | AAV97702 |
|  | AF061521 |
|  | AF232704 |
|  | AF465155 |
|  | AY849380 |
|  | BC103310 |
|  | NC\_007304 |
|  | NM\_173925 |
|  | NP\_776350 |
|  | NW\_001495205 |
|  | P79255 |
|  | Q71UR4 |
|  | S82598 |
|  | NC\_007112 |
|  | NW\_001512005 |
|  | XM\_690370 |
|  | XP\_695462 |
|  | 68354177 |
|  | 126002739 |
|  | 567083 |
|  | Dr.112992 |
|  | dre:567083 |

---

|  |  |
| --- | --- |
| Swiss-Prot ID | IL8\_HUMAN |

---

|  |  |
| --- | --- |
| Cell Localization | Secreted |
|  | Extracellular |

---

|  |  |
| --- | --- |
| Ensembl ID | ENSG00000169429 |
|  | ENSP00000306512.3 |
|  | ENST00000307407.8 |
|  | ENSP00000385908.1 |
|  | ENST00000401931.1 |
|  | ENSP00000306512 |
|  | ENST00000307407 |

---

|  |  |
| --- | --- |
| Unigene ID | Hs.624 |
|  | Hs.561078 |
|  | Hs.443948 |
|  | Hs.449585 |
|  | Hs.551925 |
|  | Hs.654584 |
|  | Bt.49740 |
|  | Dr.112992 |

---

|  |  |
| --- | --- |
| Shape | Rhomb |

---

|  |  |
| --- | --- |
| IPI ID | IPI00215621 |
|  | IPI00288914 |
|  | IPI00816576 |
|  | IPI00873454 |
|  | IPI00894389 |

---

|  |  |
| --- | --- |
| Homo sapiens Chromosome position | 4q13-q21 |

---

|  |  |
| --- | --- |
| Ensembl Transcript ID | ENST00000395775 |
|  | ENST00000307407 |

---

|  |  |
| --- | --- |
| FunctionalClass | chemokine |
|  | interleukin-8 receptor binding |
|  | protein binding |

---

|  |  |
| --- | --- |
| Bos taurus Chromosome position | 6 |

---

|  |  |
| --- | --- |
| KEGG pathway | Leukocyte transendothelial migration |

---

|  |  |
| --- | --- |
| Ensembl Gene ID | ENSG00000169429 |

---

|  |  |
| --- | --- |
| LocatorString | pmquery:prevacid, prilosec (omeprazole) 2002:2010[PDAT] |
|  | pmquery:sepsis+OR+blood+poisoning+OR+Septicemia+OR+systemic+inflammatory+response+syndrome |
|  | pmquery:curcumin prostate cancer |
|  | pmquery:egfr+transactivation |

---
